# The Vasculopathy of Juvenile Dermatomyositis

**DOI:** 10.3389/fped.2018.00284

**Published:** 2018-10-09

**Authors:** Charalampia Papadopoulou, Liza J. McCann

**Affiliations:** ^1^Infection, Inflammation, and Rheumatology Section, UCL Great Ormond Street Institute of Child Health, London, United Kingdom; ^2^Great Ormond Street Hospital NHS Foundation Trust, London, United Kingdom; ^3^Department of Pediatric Rheumatology, Alder Hey Children's NHS Foundation Trust, Liverpool, United Kingdom

**Keywords:** Juvenile, dermatomyositis, vasculopathy, antibodies, pathophysiology

## Abstract

Juvenile dermatomyositis (JDM) is a rare autoimmune disease mainly characterized by muscle and skin involvement. Vasculopathy is considered central to the pathogenesis of the disease. The exact nature of vasculopathy is not yet understood but it is a complex process with both an inflammatory and a non-inflammatory, occlusive component. Impaired function of JDM vasculature includes immune complex deposition, altered expression of cell adhesion molecules predominantly inducing Th17 cell infiltration, and endothelial cell dysfunction. Development of vasculopathy is associated with the severe extra-muscular manifestations of JDM, such as gastrointestinal and cardiac manifestations, interstitial lung disease, ulcerative skin disease or development of calcinosis, and portends a poor prognosis. Correlation of histopathological findings, autoantibodies, and extensive diagnostic workup represent key elements to the early detection of vasculopathic features and early aggressive treatment. Monitoring of vasculopathy remains challenging due to the lack of non-invasive biomarkers. Current treatment approaches provide variable benefit, but better understanding of the essential pathogenic mechanisms should help lead to improved outcomes. Whilst acknowledging that evidence is limited, this review aims to describe the vasculopathy of JDM in the context of pathophysiology, clinical features, and treatment of disease.

## Introduction

Idiopathic Inflammatory Myopathies (IIM) consist of a group of highly heterogenous diseases characterized by a systemic inflammatory process. Muscle weakness and skin involvement are common characteristics but other organs can also be affected (1). Juvenile Dermatomyositis (JDM) is the commonest childhood IIM seen in ~85% of cases, while polymyositis consists of <5% of the pediatric IIM cases (2). The reported annual incidence of JDM ranges between two to four cases per one million children per year (2). It is more common in females compared to males, with reported female: male ratios ranging between 2:1 and 5:1 in different cohorts (2).

The exact etiology of JDM is not fully understood and is thought to be multifactorial. The prevailing hypothesis is that JDM is the result of genetic susceptibility and environmental triggers, which subsequently cause dysregulation and dysfunction of the immune system resulting in tissue inflammation (3). Vasculopathy seems to play a central role in the pathogenesis of myositis and cutaneous manifestations (4), but is also central to other severe systemic features that contribute significantly to the burden of disease in children. The vasculopathy associated with JDM is thought to underlie the development of intestinal ischaemia and perforation, cutaneous ulceration, interstitial lung disease, and calcinosis (5, 6).

The identification of vascular involvement in JDM is challenging, and depends on extensive immune, histopathologic and imaging diagnostic approaches together with clinical experience and expertise. It may influence choice of therapeutic strategy and prediction of individual patient prognosis. Therefore, to better understand the pathophysiology, histopathological findings, clinical manifestations, and associated complications is of great clinical significance.

## Pathophysiology

**Figure 1:**
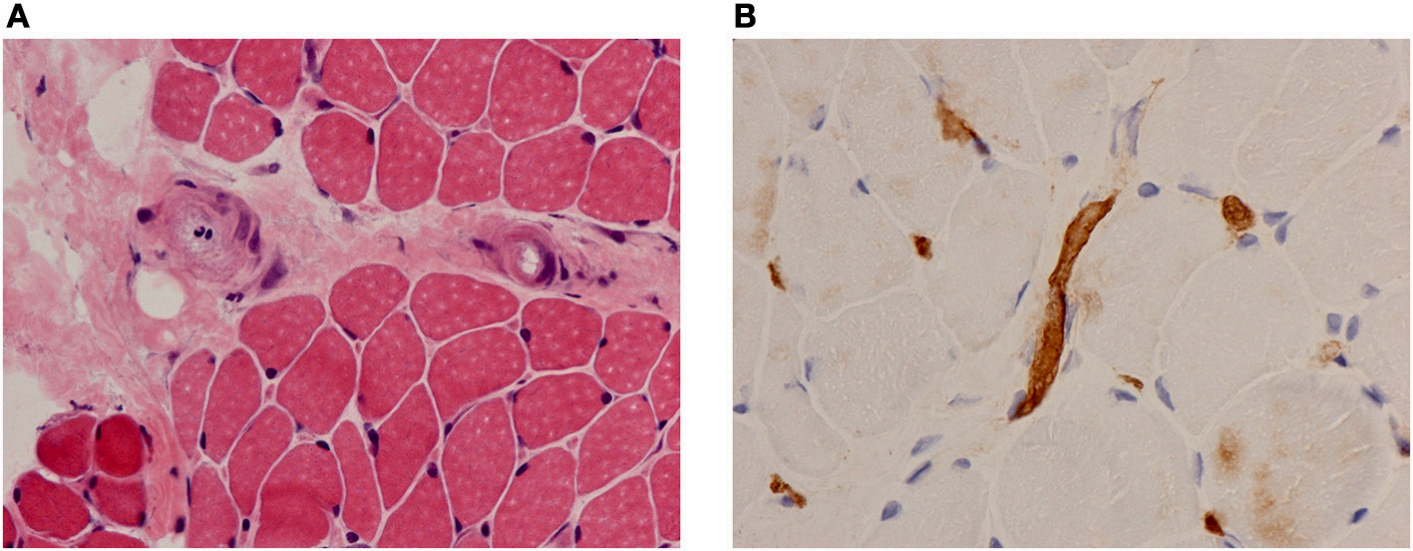
Muscle biopsy findings in a patient with JDM. **(A)** Haematoxylin and eosin (H&E) stain showing vessel endothelial swelling. **(B)** Immunohistochemical staining for membrane attack complex (MAC) showing deposition on muscle capillaries. Magnification 40x.

The exact nature of vasculopathy remains unclear and is likely to change at different stages of the disease. Early on there is evidence of a true inflammatory small vessel vasculitis driven by interferons and other cytokines (4, 7); but also a later, non-inflammatory occlusive vasculopathy with capillary drop out (5, 8). Necrotising capillaritis is seen with complement deposition in lesional muscle biopsies suggestive of a small vessel vasculitis mediated by immune complexes (4, 9) (Figures 1A,B). Immunoglobulin and complement have been identified in the vessel walls of skeletal muscle in children with JDM (4). Endothelial cells in the affected muscle produce IL-1 along with other cytokines (10) and promote inflammation through paracrine upregulation of endothelial intercellular adhesion molecule-1 (ICAM-1) and vascular cell adhesion molecule-1 (VCAM-1) (11). Moreover, activated endothelium expresses binding sites for various chemokines, resulting in attraction of other inflammatory cells, and has an important effect on angiogenesis. Angiostatic chemokines are expressed at high levels in biopsy specimens of untreated JDM patients, correlating with the degree of capillary loss and mononuclear cell infiltration (12). Neovascularization may then occur later in the disease process (13).

Activated dendritic cells present within lesional muscle release interferons which have a range of biological effects on the endothelium, such as increased expression of major histocompatibility complex (MHC) class I and class II and adhesion molecules promoting T-cell migration (7, 12, 14, 15), such as macrophage inflammatory protein (MIP), monocyte chemoattractant protein (MCP)-1, and MCP-2. Involvement of T cells occurs via lesional T helper type 17 (Th17) cells. The participation of Th17 cells also seems to lead to induction of IL-6 and IL-17, which correlate with the interferon response and with active disease. Indeed, interferons are considered central to the pathogenesis of JDM and important drivers of the associated vasculopathy (7).

Later in the course of JDM, the vasculopathy is characterized by endothelial cell swelling, necrosis, and luminal occlusion of capillaries and arterioles (5, 8). Another common and well documented observation in JDM is capillary drop-out, which is the predominant vasculopathic feature observed in muscle biopsies and in the skin (8, 16, 17). Occlusive vasculopathy also contributes significantly to many of the severe late sequelae of JDM including cutaneous ulceration, intestinal ischaemia, and ultimately gut infarction (18). It is possible that occlusive vasculopathy contributes to chronic subcutaneous calcinosis (19).

The vasculopathy of JDM may present with different clinical phenotypes. The extent of symptoms includes mild forms limited to cutaneous vessels, and severe, life-threatening manifestations with organ involvement. The following section addresses the pathophysiology and clinical manifestations of disease relating them where possible to vasculopathy.

### Cutaneous manifestations

**Figure 2:**
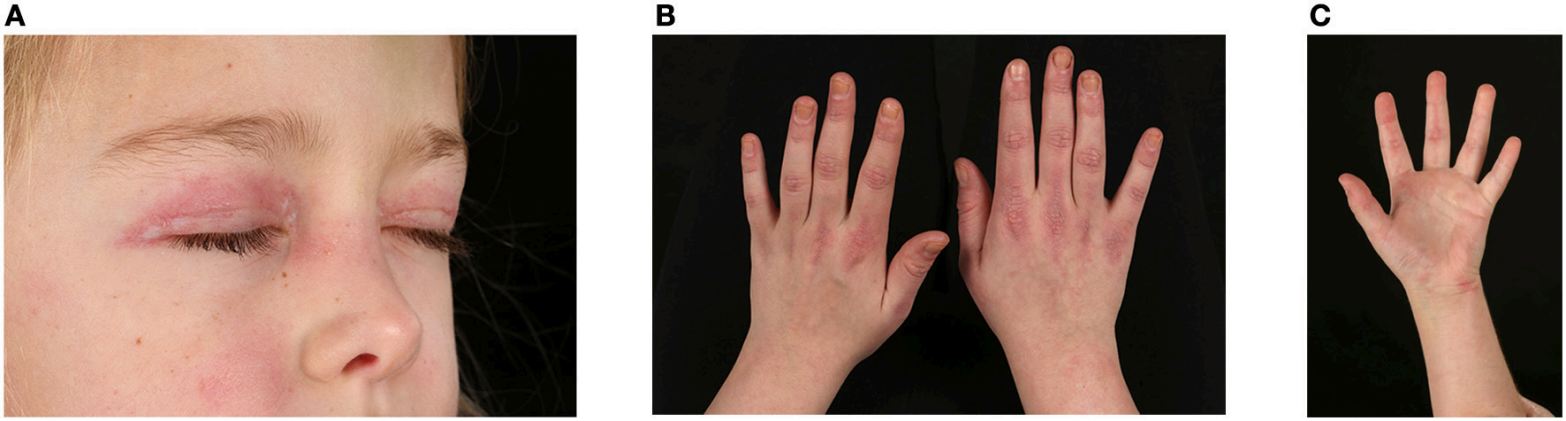
Skin changes seen in juvenile dermatomyositis. **(A)** Heliotrope rash – erythema involving both upper eyelids in a patient with JDM. Written informed parent consent was obtained for the publication of this image. **(B)** Gottron's papules over metacarpal and interphalangeal joints with linear extensor erythema. **(C)** Palmar vasculopathy- palmar erythema, most prominent over the joint creases.

JDM is associated with a wide range of skin rashes, illustrated and described in detail by Dugan et al. (20), but most pathognomonic is the heliotrope rash involving the upper eye-lids and Gottron's papules over the small joints of the hands and large joints (Figures 2A,B). Periungual erythema and nailfold capillary loop changes are often present and palmar erythema can be seen (20) (Figure 2C). Skin ulceration is a serious manifestation of JDM that can be life threatening. Ulcers presumably mirror significant vasculopathy in the skin, caused by hypoxia and ischaemia of the affected tissues and may signal vasculopathy in other organs (i.e., intestinal ischaemia and perforation, pulmonary fibrosis and interstitial lung disease) (21, 22). Patients with ulcerative skin disease are considered to have a more severe disease course with a worse long-term prognosis (23). Vasculitic ulcers are present in 23–30% patients (24) and can be seen in particular at the corner of the eyes (usually heal leaving a chicken-pox-like scar), and over elbows or other pressure points (5). Periorbital or generalized edema has been reported in 32% of JDM cases in the UK cohort (25); usually present at diagnosis or during significant disease flare. It can relate to a more severe disease course and indicate resistance to treatment (26). It is considered the result of a generalized capillary leak due to damage of the vascular endothelium (26).

Dystrophic calcification of tissues occurs in 20–40% of patients with JDM, although seen much less frequently in adults with DM (19). Calcinosis can be complicated by ulceration of the overlying skin, contractures of the joints when crossing joint margins (27), pain due to entrapment neuropathy, or topical inflammation with redness, tenderness, and swelling (24, 28). Nailfold capillary changes seen in JDM include dilatation, occlusion, bushy loops, hemorrhages, and capillary drop-out (29), and are present in 80–91% of children at the time of diagnosis. Nailfold capillaroscopy is a non-invasive technique that provides quantitative information about the loss of capillary end-row loops (ERL), areas of decreased or absent vascularity, and formation of arboreal loops (30). Visualization of the nailfolds can be easily performed at the bedside with the use of a light and magnification from a dermatoscope, ophthalmoscope, or auroscope with or without water soluble gel (24). The skin around the nail beds can be erythematous (peri-ungual erythema) and sensitive. Cuticular overgrowth can be seen. Nailfold changes correlate with prolonged disease course, overall disease activity, skin disease activity, and poor response to treatment (30, 31). A similar vessel pattern has been observed in the marginal gingiva that could be part of the vasculopathy associated with JDM (32).

### Cardiac involvement

Recent case-control echocardiographic studies in children with JDM have suggested that up to 25% of patients have evidence of subclinical left ventricular diastolic dysfunction, and a high prevalence of pathological electrocardiographic (ECG) abnormalities (33). Children with JDM have also been found to have reduced heart rate variability which was associated with impaired myocardial function suggestive of reduced cardiac vagal control (34). Whilst studies in children are limited, Rosenbohm et al. demonstrated signs of subclinical myocardial inflammation on cardiac MRI scanning in ~40% of patients with adult onset inflammatory myositis (35), while pericarditis has also been reported (36). Hypertension is seen in 25 to 50% patients probably attributed to both the microvasculopathy of the disease but also corticosteroid treatment (31). In a recent study of adults with juvenile onset DM, subclinical cardiovascular disease and increased carotid intima-media thickness was demonstrated suggestive of early onset atherosclerosis despite their young age and absence of other traditional cardiovascular risk factors (37). Persistence of skin disease activity was shown to correlate with persistence of cardiac dysfunction in a small follow-up study, suggesting that a common vasculopathic process affects the skin and myocardium (30). The significance of this subclinical cardiac involvement and potential contribution to cardiovascular morbidity is currently unknown.

Accelerated atherosclerosis has been demonstrated as an important factor of mortality and morbidity in patients with autoimmune diseases (38). A recent study comparing patients with rheumatoid arthritis and patients with diabetes found equal frequency of atherosclerosis between the two diseases (39). Regardless of the exact nature of small vessel vasculopathy in JDM, in the longer term there may be a generalized secondary systemic vasculopathy affecting larger arteries, ultimately leading to accelerated atherosclerosis and premature cardiovascular morbidity later in adulthood. This could be the result of the primary chronic vasculopathic nature of the disease causing “polyangiitis overlap,” and/or uncontrolled chronic systemic inflammation, as in other autoimmune diseases (40). In support of this, studies of adults with dermatomyositis demonstrate an almost twice-higher risk of acute myocardial infarction and stroke when compared to the general population (41, 42). Interestingly, a small pilot study compared carotid intima-media thickness (CIMT) and flow-mediated dilatation (FMD) as surrogate markers of atherosclerosis in 8 adults with a history of JDM and revealed increased CIMT in JDM patients compared to 8 healthy controls despite the young age (37). Moreover, recently a large retrospective study demonstrated that JDM was associated with higher odds of cardiovascular and cerebrovascular disease in adolescents, including atherosclerosis, transient ischaemic attacks and cerebral infarction (43). It is thus possible that the combination of chronic endothelial injury caused by persistent small vessel vasculitis, chronic systemic inflammation, long-term corticosteroid use, sedentary lifestyle, and conventional cardiovascular risk factors predispose patients with JDM to early atherosclerosis.

### Gastrointestinal tract involvement

Gastrointestinal tract involvement occurs in 5–37% of JDM cases (24). This includes dysphagia, bowel dysmotility, vasculitis with associated malabsorption, and other more severe features of gastro-intestinal vasculopathy that can be life threatening. Vasculopathy in the gastrointestinal tract may present with abdominal pain, rectal bleeding, intestinal ischaemia, pneumatosis, and finally perforation (44). Acute inflammatory vasculitis and chronic gastrointestinal occlusive arteriopathy have been described in JDM patients, indicating that the underlying pathology is complex and multifactorial (21). Initially inflammatory cells seem to infiltrate the gastric mucosa and play a significant role in pathogenesis. Persistent severe abdominal pain is a worrying sign that warrants prompt investigation and radiographic imaging. The main radiographic finding is thickening of the bowel mucosal folds demonstrated by barium swallow and follow-through studies (45).

### Pulmonary vasculitis

Pulmonary involvement is much less common in children with JDM than adult-onset IIM, but interstitial lung disease (ILD) may still complicate childhood cases (46). Connective tissue disease associated-ILD is thought to be initiated by microvascular injury leading to endothelial cell damage and alveolar epithelial injury (47) leading to the release of numerous cytokines and growth factors which play a key role in the development of lung disease. The exact mechanisms are still not fully understood. Abnormal pulmonary function tests are recorded in more than half of JDM cases (48). In particular, diffusion capacity may be decreased at early stages of ILD. Progression can be rapid and may be life-threatening unless treated aggressively (49). High serum anti-melanoma differentiation-associated gene 5 (MDA5) antibodies and anti-synthetase antibodies are associated with rapidly progressive interstitial lung disease. Typically, ILD presents with increasing cough and progressive dyspnoea, although it may be asymptomatic. Diffuse alveolar hemorrhage (DAH) and pneumomediastinum are rare but life-threatening manifestations of JDM (50–52). Interstitial fibrosis, vasculitis and infarction have been suggested as possible mechanisms in adults with DM (53).

### Other organ involvement

Although rare, vasculopathy of the central nervous system has been reported in children with JDM (54, 55). It can be very difficult to diagnose this life-threatening complication. It most commonly presents with hallucinations and seizures, and MRI/MRA can be useful diagnostic tools (55). Most reported cases had active retinal vasculitis (54, 55) suggesting that the eyes represent another organ that can be affected by JDM vasculopathy. However, a retrospective review of 82 patients at a single center demonstrated that retinopathy was rare and concluded that routine assessment was not warranted for patients without visual symptoms (56).

## Myositis specific antibodies

Myositis specific antibodies (MSA) and myositis associated antibodies (MAA) are present in at least 60% of JDM cases (57). Different types of antibodies are associated with specific clinical manifestations but phenotype may differ according to the age of the patient at time of disease onset (57). The commonest MSAs in JDM patients include anti-TIF1γ, anti-NXP2 and anti-MDA5. Anti-TIF1γ (p155/140) is reported in 23–29% of patients with JDM and is associated with more severe skin disease, development of skin ulceration and a long disease course (58), while anti-NXP2 (p140) is mainly connected with the development of calcinosis in pediatric cases (59). Anti-MDA5 antibodies are associated with mild myositis, arthritis mainly of the small joints, ulceration of the skin and increased risk of the disease to be complicated with interstitial lung disease (60). Recently, a number of anti-endothelial cell antibodies (AECA) have been identified in the plasma of JDM cases (61), which are typical of vasculitis and vascular thrombosis in lupus patients (62). Their clinical and diagnostic significance remains unclear and needs to be further elucidated. It is not known if specific antibodies pre-exist the development of the specific disease features with which they are associated; if so, they could be useful as prognostic biomarkers and guide further therapeutic management (57). In support of this, Deakin et al. demonstrated that MSA in combination with muscle biopsies severity scores can be predictive of long-term treatment status and prognosis in children with JDM (63).

## Treatment

The intensity of initial therapy is determined by the severity of the presenting symptoms (64) including the presence of life-threatening weakness, major organ involvement, ulcerative skin lesions or extensive calcinosis. To date, management approaches for JDM cases have been mainly based on small case series and anecdotal experience as only a few randomized clinical trials exist to guide decisions. In an attempt to standardize treatment strategies, several therapeutic algorithms for the treatment of JDM have been published based on expert consensus in North America and Europe (65–69). The Childhood Arthritis and Rheumatology Research Alliance (CARRA) have developed Consensus Treatment Plans (CTPs) for the initial treatment of JDM (69), treatment after the first 3 months (68), treatment of skin predominant disease (65), and persistent skin rash (66). It is important to highlight that these CTPs do not consist of therapeutic guidelines *per se*, but they represent arms for proposed comparative effectiveness studies, developed via consensus methodologies. Moreover, evidence-driven consensus-based recommendations for diagnosis and treatment of JDM have also been published as part of a European initiative called Single Hub and Access point for pediatric Rheumatology in Europe (SHARE) (67).

Delayed or inadequate treatment has been associated with poorer prognosis and early aggressive treatment has been demonstrated to improve the long-term outcome (70). Pharmacological interventions for JDM include corticosteroids, disease-modifying drugs (DMARDs), and biologics agents. Different steroid regimes have been proposed for the initial treatment of JDM with the most commonly used being oral prednisolone or intravenous (IV) pulses with methylprednisolone. IV methylprednisolone is preferred for patients with evidence of active vasculopathy as it has been demonstrated that the absorption of oral prednisolone may be decreased in children with evidence of loss of nail-fold capillary ERL by capillaroscopy (71). The exact role of the two different regimes in the treatment of JDM needs to be further elucidated (72). Regardless of the regime used, corticosteroid dose is then tapered over a period of months depending on the response. Methotrexate (MTX) has become the first line treatment of JDM, recommended in combination with corticosteroids at time of diagnosis (66). Ciclosporin has also been used in many centers as a steroid-sparing agent. In a recent randomized controlled trial (RCT) where monotherapy with corticosteroids was compared with the combination of prednisone with either MTX or ciclosporin, combination therapy had greater efficacy than corticosteroid alone. More adverse effects were seen with ciclosporin than MTX, supporting the usual practice of corticosteroid and MTX as first line treatment (73). Intravenous immunoglobulin (IVIG) is recommended for patients with JDM refractory to steroids and methotrexate (67, 68). An alternative DMARD suggested for severe/refractory cases of JDM or where methotrexate is not tolerated, is mycophenolate mofetil (67), beneficial for both muscle and skin disease. MMF has also been shown to be effective in adult patients with myositis associated interstitial lung disease (ILD) not responding well to steroid treatment (74). Azathioprine is less commonly used in JDM (75).

Several therapeutic agents have been proposed for the treatment of severe/refractory JDM cases. A randomized trial that included 152 adults with myositis and 48 children with JDM demonstrated that 83% of patients met the definition of improvement on rituximab (76), even though the study failed to reach its primary or secondary endpoints possibly due to limitations concerning the trial design (77). The overall response rate, steroid-sparing effect and re-treatment response suggests that rituximab has a beneficial effect, particularly in children and those with antibody positive disease (76). In 2008, a case series reported the efficacy of infliximab in children with refractory JDM and development of calcinosis (78). Evaluation of 66 JDM patients, recruited from the UK JDM Cohort and Biomarker Study and actively treated with anti-TNF agents, both infliximab and adalimumab showed significant improvements in overall disease activity, including both muscle and skin involvement (79). In contrast, etanercept failed to demonstrate efficacy in two prospective case studies in adult and juvenile dermatomyositis, respectively (80, 81). There have been reports of TNF inhibitor induction or exacerbation of DM (82, 83) with the exact pathophysiological mechanism not yet understood. Abatacept, a soluble fusion protein comprising the extracellular domain of human cytotoxic T-lymphocyte associated antigen 4 (CTLA-4) and a fragment of the Fc domain of human immunoglobulin G1, has been reported to be effective in a recalcitrant JDM case with ulcerations and calcinosis (84) and a clinical trial (*NCT number:* NCT02594735) is currently underway.

Cyclophosphamide (CyC) is another third line therapeutic agent mainly reserved for JDM cases refractory to most other therapies and for cases with significant organ involvement such as cardiopulmonary involvement, skin ulceration or gastrointestinal vasculopathy. Several case studies demonstrate the efficacy of treatment with CyC in both pediatric and adult patients with IIMs (74, 85). A recent analysis of a UK cohort of JDM patients treated with CyC indicated good efficacy and low rates of adverse events (86).

As JDM is now increasingly recognized as a condition that falls into the category of diseases driven by interferons (87), JAK inhibition has been suggested as a potential new targeted therapeutic regime in refractory cases of adult dermatomyositis (88).

## Conclusions

JDM represents the commonest idiopathic inflammatory myopathy of childhood. When looking at affected tissues, vascular and perivascular inflammation is a predominant feature of the disease, thus early descriptions recognize JDM as a systemic angiopathy of the childhood. The presence and persistence of vascular involvement is associated with more severe and refractory disease and the development of life-threatening complications. With recent therapeutic strategies, survival, and outcomes of JDM have improved significantly with a reported mortality of <2% (89), but the long-term outcome differs substantially between patients. The exact pathophysiology of JDM vasculopathy remains unclear and may change over the course of disease. Complement deposition and endothelial cells activation with pronounced expression and activation of adhesive molecules are considered key factors in the pathogenesis of the disease, while the role of autoantibodies needs to be further elucidated. Early recognition of vasculopathic features and early aggressive treatment are key components to a better outcome. Predicting and monitoring the development of vasculopathy throughout the disease course remains challenging. Current therapeutic regimes still lack specificity and in severe cases fail to control disease activity. Targeting candidate immune pathways that may be contributing to disease pathogenesis is an active area of research and an unmet need.

## Author contributions

CP and LM have contributed to the draft and made significant intellectual contribution to the generation of the final manuscript.

### Conflict of interest statement

The authors declare that the research was conducted in the absence of any commercial or financial relationships that could be construed as a potential conflict of interest.
